# Light-induced magnetoresistance in solution-processed planar hybrid devices measured under ambient conditions

**DOI:** 10.3762/bjnano.8.150

**Published:** 2017-07-21

**Authors:** Sreetama Banerjee, Daniel Bülz, Danny Reuter, Karla Hiller, Dietrich R T Zahn, Georgeta Salvan

**Affiliations:** 1 Semiconductor Physics, Technische Universität Chemnitz, Germany; 2 Center for Microtechnologies, Technische Universität Chemnitz, Germany; 3 Fraunhofer ENAS, Chemnitz, Germany

**Keywords:** HED-TIEs, hybrid electronic devices, organic field-effect transistors (OFETs), organic magnetoresistance, planar hybrid devices, TIPS-pentacene

## Abstract

We report light-induced negative organic magnetoresistance (OMAR) measured in ambient atmosphere in solution-processed 6,13-bis(triisopropylsilylethynyl)pentacene (TIPS-pentacene) planar hybrid devices with two different device architectures. Hybrid electronic devices with trench-isolated electrodes (HED-TIE) having a channel length of ca. 100 nm fabricated in this work and, for comparison, commercially available pre-structured organic field-effect transistor (OFET) substrates with a channel length of 20 µm were used. The magnitude of the photocurrent as well as the magnetoresistance was found to be higher for the HED-TIE devices because of the much smaller channel length of these devices compared to the OFETs. We attribute the observed light-induced negative magnetoresistance in TIPS-pentacene to the presence of electron–hole pairs under illumination as the magnetoresistive effect scales with the photocurrent. The magnetoresistance effect was found to diminish over time under ambient conditions compared to a freshly prepared sample. We propose that the much faster degradation of the magnetoresistance effect as compared to the photocurrent was due to the incorporation of water molecules in the TIPS-pentacene film.

## Findings

The field of molecular spintronics has received lot of research interest in the last years because of the possibility of designing nano-scalable molecule-based multi-functional devices [1]. Among the various reports on spin transport and/or magnetoresistive measurements in molecule-based devices, those regarding organic magnetoresistance (OMAR) are particularly interesting, because this effect can be observed at room temperature and in low magnetic fields of several milliteslas [2].

Several organic semiconductors consisting of small molecules such as aluminium-tris(8-hydroxyquinoline) (Alq_3_) [3–4], pentacene [5], α-sexithiophene [6] or even conjugated polymers such as poly(*N*-vinyl carbazole) and poly (*p*-phenylene vinylene) [6–7] derivatives have been found to exhibit an OMAR effect as an intrinsic material property without any spin injection from ferromagnetic materials. Even though OMAR has been extensively investigated over the last few years, most of the studies on OMAR have been carried out mainly on vertical devices, in which the active organic layer is sandwiched between two conductive metal layers [3–4,8]. Saragi et al. carried out studies on the magnetic field effect on three terminal bottom-contact organic field-effect transistors (OFET) and showed the existence of light-induced magnetoresistance in pentacene and its derivative 6,13-bis(triisopropylsilylethynyl)pentacene (TIPS-pentacene) molecules [5,9] deposited by thermal evaporation in vacuum. The influence of gate voltage on the magnetoresistance was also thoroughly investigated in these two reports.

In fact, most of the studies on OMAR in small molecules reported so far deal with thermally evaporated molecules and were carried out in a controlled atmosphere such as nitrogen-purged glove boxes or a cryostat to prevent the degradation of the devices in ambient atmosphere [3,5,9].

Here we demonstrate that light-induced magnetoresistance can be observed at room temperature even in ambient atmosphere. TIPS-pentacene is an ideal candidate for this proof of concept, as it is a solution-processable molecule that exhibits high carrier mobility and air-stability [10–11]. Moreover, the existence of light-induced magnetoresistance in TIPS-pentacene was already shown for thermally evaporated films by Saragi et al. [9]*.*

In order to investigate the influence of the electrode distance on the OMAR magnitude and on the timeline of the OMAR response to the switching of magnetic field, we used two planar device geometries: i) commercially available micro-structured bottom-contact OFET substrates as used in [5] to compare the results obtained from solution processed devices with the previously reported results for evaporated TIPS-pentacene and ii) submicrometre-structured substrates for hybrid electronic devices with trench isolated electrodes (HED-TIE) designed and developed in this work.

Negative magnetoresistance (positive magnetoconductance) was observed for both types of devices similar to the observations in [9]. Magnetoresistance is defined as the change in resistivity of a material/device due to application of an external magnetic field. Magnetoconductance (MC) is the change in conductivity of a material/device upon application of an external magnetic field. The magnetoresistance effect, however, was found to degrade over time significantly faster than the photocurrent. We suggest that this degradation is due to incorporation of water molecules from the ambient atmosphere into the TIPS-pentacene.

**Figure 1:**
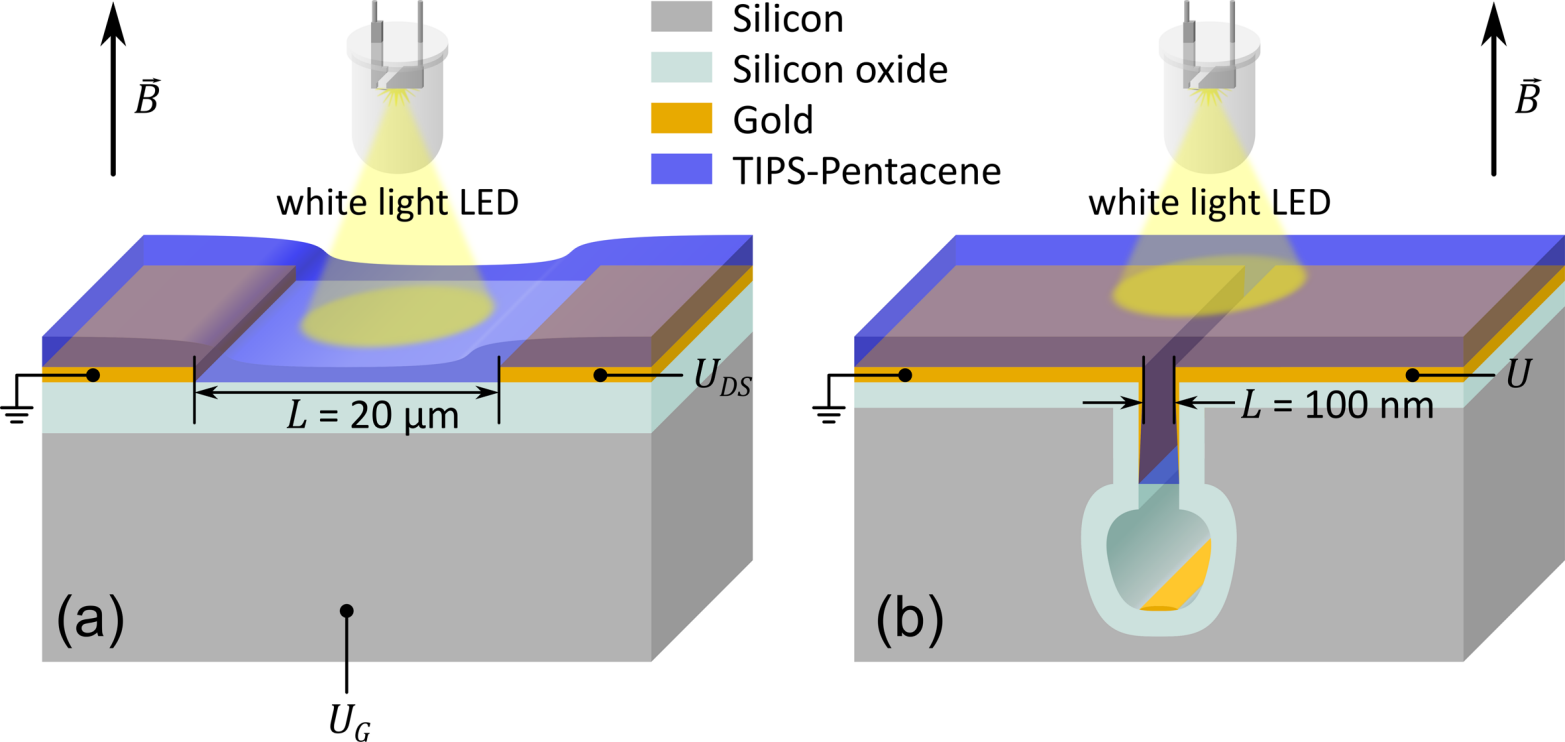
Schematic diagram of the experimental setup: (a) Commercial bottom-contact OFET substrates; (b) planar device structure with trench-isolated electrodes (HED-TIE).

The OFET substrates were purchased from Fraunhofer IPMS (Dresden, Germany) with channel lengths varying between 2.5 µm and 20 µm and a channel width of 10 mm. The gate oxide SiO_2_ is 270 ± 10 nm thick and the source and drain electrodes are made of 30 nm Au with a 10 nm ITO adhesion layer. It should be noted that the gate electrode was kept at ground voltage for the magnetoresistance measurements and the devices were used as two-terminal devices for making the results comparable with the HED-TIE devices. For the HED-TIE devices, submicrometre trenches were patterned using conventional UV lithography and partially refilled with a thermally grown SiO_2_ layer of 130 nm. The cavity-like trench (see Figure 1b for a sketch) electrically isolates the electrodes. The process flow for the preparation of the HED-TIEs is similar to that described in [12]. 30 nm Au was deposited as the electrode layer with a 10 nm Cr as the adhesion layer (please refer to Supporting Information File 1 for further details of HED-TIE structures). TIPS-pentacene powder with a purity of 99.9% was purchased from Ossila. The solution was prepared in a mixture of toluene/tetralin (2:1) with a concentration of 8 mg/mL. Prior to drop-coating, the fabricated structures with gold electrodes were cleaned using acetone, ethanol and deionized water respectively. 5 µL of solution was used to drop-coat the OFET substrates of 0.8 cm × 0.8 cm area and 3 µL solution was used to drop-coat the HED-TIE devices for a 1 cm × 1 cm area. TIPS-pentacene solution coats the gold surface much better than the SiO_2_ surface and hence to eventually have similar coverage at the device-channel area, different amounts of solution were used for OFET and HED-TIE devices. The drop-coating of the substrates was performed at 65 °C on a hot plate and the samples were kept at 65 °C for 30 min after drop-coating, to initiate crystallization of the TIPS-pentacene film and to ensure evaporation of the solvents from the film. Unless otherwise mentioned, the electrical characterisation including the photoswitching, *I–V* characteristics and magnetoresistance measurements was performed immediately after the sample preparation. The total measurement time was about 1 h.

The schematic diagram of the experimental set-up is shown in Figure 1. The entire sample surface was illuminated with a white light emitting diode (LED), with an emission ranging from 400 to 700 nm for the measurements of the photocurrent and of the magnetoresistance. The measurements were performed at room temperature (ca. 25 °C) and the ambient humidity was maintained at (20 ± 5)%.

The electrical measurements were conducted using a Keithley 2636A SYSTEM source-meter unit in the auto-range mode. The magnetic field was applied perpendicular to the electrical transport channel and to the substrate plane, using an electromagnet. It should be mentioned, though, that previous reports showed OMAR to be independent of the sign and direction of the applied magnetic field [13].

**Figure 2:**
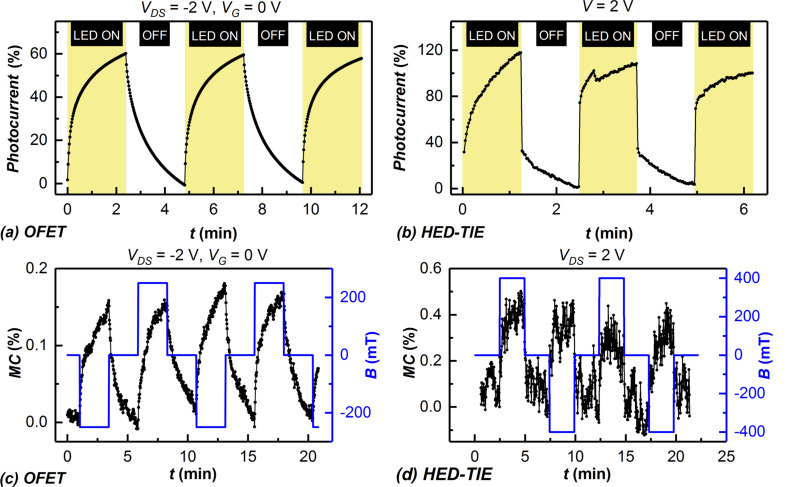
Light-switching behaviour of TIPS-pentacene-based (a) organic field-effect transistor (OFET) and (b) hybrid electronic device with trench-isolated electrodes (HED-TIE). Light-induced magnetoresistance in TIPS-pentacene obtained from both types of planar devices: (c) OFET and (d) HED-TIE device.

Figure 2a and Figure 2b show the percentage of photocurrent with respect to the dark current, when the white light LED was switched on and off for OFET and HED-TIE devices, respectively. The percentage of the change in current was found to be more representative when comparing two device architectures with significantly different volume of the active organic channel. The photocurrent of the HED-TIE devices is a factor of two higher and the photo-switching occurs to be faster in the HED-TIE devices, due to the smaller electrode gap.

Figure 2c and Figure 2d show the magnetoconductance response of the same devices. Here, the device current over time was measured by switching on and off a constant applied magnetic field while the illumination was kept on throughout the measurements. Both OFETs and the HED-TIE devices were found to exhibit positive magnetoconductance (negative magnetoresistance) as previously reported for evaporated TIPS-pentacene [9]. The magnitude of OMAR is independent of the sign of the applied magnetic field, as expected.

For the switching of both light and magnetic field, the HED-TIE devices were found to respond faster whereas the OFETs exhibit a slower carrier relaxation. This can be attributed to higher electric fields and shorter charge-carrier transit times in the shorter channels of the HED-TIEs (ca. 100 nm) compared to that of OFETs (20 µm). Also the magnitude of switching in the HED-TIE device for both light and magnetic field was found to be twice as much as OFET, similar to the photocurrent.

It should be mentioned here that the TIPS-pentacene OFETs fabricated by thermal evaporation in [9] showed a fast switching response, similar to our HED-TIEs. This is most probably due to the significantly smaller volume of the OFET channel (78 nm film thickness in [9], compared to ca. 500 nm film thickness in our drop-coated OFETs at the same channel length), because the mobility values determined for our solution-processed OFETs are in the range from 10^−2^ to 1 cm^2^·V^−1^·s^−1^, which is higher than the mobility values reported for thermally evaporated TIPS-pentacene (6·10^−3^ to 1 cm^2^·V^−1^·s^−1^) [9].

As no magnetoresistance was observed in the absence of illumination and hence of a photocurrent, the observed effect in the solution-deposited TIPS-pentacene OFETs and HED-TIEs is supposedly based on the presence of spin-carrying polarons related to electrons and holes created by photoexcitation. These weakly interacting polarons are labelled as “electron–hole (e–h) pairs” and are considered to contribute to OMAR because of their flexible spin configuration (singlet or triplet). The same mechanism was proposed to be responsible for the OMAR observed in thermally evaporated TIPS-pentacene OFETs in [9], which is an indication that the processing methods of the TIPS-pentacene do not influence the intrinsic mechanism of the OMAR in this organic semiconducting material. In the e–h model, the recombination into electrically neutral excitons and the dissociation of the weakly bound e–h pairs into charge carriers available for the electrical transport is influenced by an applied magnetic field, in favour of the latter effect [9,14]. An applied magnetic field will thus trigger an increase in the conductance of the device [9,14]. It should be noted that although the applied constant magnetic field was different for our OFETs and HED-TIEs, this should not have an influence on the magnitude of the OMAR effect. It was shown in [5] for pentacene that the magnitude of OMAR almost reaches a saturation at about 80 mT and a similar saturation field was also reported for other materials such as Alq_3_ [3].

**Figure 3:**
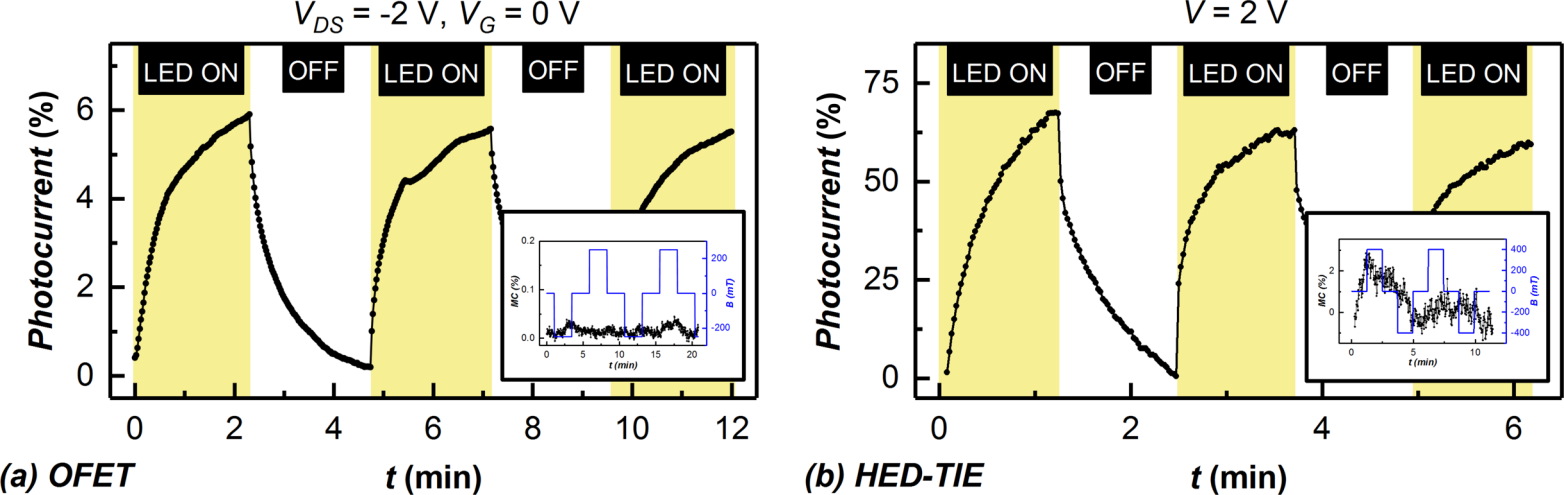
(a) Light switching of an OFET prepared by drop-coating of TIPS-pentacene diluted in water. The inset shows absence of the magnetoresistance in such an OFET. (b) Degraded light-switching behaviour of a TIPS-pentacene HED-TIE after two days of exposure to ambient atmosphere. The inset shows the response to applied magnetic field.

The observed magnetoresistance effect was found to diminish approximately after two days of sample preparation in case of the HED-TIE devices kept under ambient conditions, while the photocurrent decreased from 120% to only 65%. The photoresponse time, however, was increased compared to the freshly prepared sample (shown in Figure 3b). The magnetoresistive effect and the photocurrent in the OFETs were also found to decrease in magnitude, but the magnetoresistance did not disappear completely in the same time interval considered for the HED-TIEs. This could be due to the TIPS-pentacene layer being thicker for the OFETs, which confers a higher stability over time. The degradation of the devices in ambient atmosphere can be attributed to the penetration of oxygen and/or water molecules in the TIPS-pentacene film, additionally accelerated by white light illumination. Vollmer et al. showed [15], by investigating the occupied electronic states using ultraviolet photoemission spectroscopy, that the diffusion of molecular oxygen and water from the ambient into pentacene layers is reversible when the air exposure takes place in the dark or under visible light. The exposure to ambient under UV illumination leads to a reaction, most probably with singlet oxygen and/or ozone [15]. As the magnetoresistance measurements are performed in presence of white light with a spectrum ranging from 400 to 700 nm, we believe that different parts of the wavelength spectra can have different contributions to the device degradation. It was also shown by Jurchescu et al. [16] that pentacene single crystals behave differently when the electrical conductivity is measured in ambient or dry air, with and without illumination. It should be mentioned here that previously we found the decrease in photoluminescence caused by the diffusion of oxygen/water in TIPS-pentacene films in the HED-TIE devices to be reversible [12] and that the recovery is much faster under nitrogen purging and single-wavelength illumination.

To verify the role of the diffusion of water molecules into the TIPS-pentacene film in the deterioration of the magnetoresistance, an OFET was prepared by drop-coating a substrate with the same TIPS-pentacene solution as used for the preparation of the devices discussed before (cf. Figure 2) but now diluted (1:1) in deionized water. As shown in Figure 3a, the magnitude of the photocurrent of the freshly prepared device was found to be only 6% (compared to 60% for the device prepared with a water-free solution, see Figure 2a) whereas the magnetoresistance disappeared completely in this case. The output characteristics of such a device showed (please refer to Supporting Information File 1) a drastic change in the line shape compared to the TIPS-pentacene transistor prepared from water-free solution.

This experiment shows that TIPS-pentacene, despite of its solution processability in non-polar solvents, exhibits a degradation in electrical properties upon water incorporation in the films. We assume that the water molecules in the film shift the energy level of the singlet and triplet states of the e–h pairs and thereby change the probability of intermixing between these two states. In a changed energy landscape of the excited electronic states, the effect of the magnetic field on the singlet/triplet intermixing could become negligible, explaining the dramatic reduction in magnetoresistance.

To summarize, we demonstrated the presence of light-induced negative magnetoresistance in freshly prepared solution-processed TIPS-pentacene planar devices of two different architectures, measured under ambient atmosphere. The HED-TIE devices with smaller electrode gap yielded a higher photocurrent, a higher magnetoresistance and a lower switching time. Although TIPS-pentacene is supposed to be stable in air, the influence of ambient atmosphere was found to cause diminishing of the magnetoresistance of the fabricated devices within a period of two days after sample preparation. We assume this is due to incorporation of water molecule in the TIPS-pentacene film when it is exposed to ambient. To support this hypothesis, we showed the absence of magnetoresistance in OFETs prepared with TIPS-pentacene solution diluted in deionized water accompanied by a reduction, but not disappearance of the photocurrent. We propose that the usage of a suitable capping layer could protect the organic film and thus increase the lifetime of such devices, if used as magnetoresistive sensors working under ambient atmosphere.

## Supporting Information

Supporting Information describes the fabrication of HED-TIE devices and the electrical output characteristics for both HED-TIE and OFET devices.

File 1 Additional epxerimental data.
