# Supplementary material for: Light-induced magnetoresistance in solution-processed planar hybrid devices measured under ambient conditions
Source: Beilstein J Nanotechnol. 2017 Jul 21;8:1502–7. doi: 10.3762/bjnano.8.150 (PMC5530628; doi:10.3762/bjnano.8.150)
Supplement: File 1 — Additional epxerimental data. [file Beilstein_J_Nanotechnol-08-1502-s001.pdf]

Supporting Information

for

Light-induced magnetoresistance in solution-processed planar hybrid devices measured under ambient conditions

Sreetama Banerjee*[1,2], Daniel Bülz[1], Danny Reuter[2,3], Karla Hiller[2], Dietrich R. T. Zahn[1] and Georgeta Salvan*[1]

Address: [1]Semiconductor Physics, Technische Universität Chemnitz, Germany; [2]Center for Microtechnologies, Technische Universität Chemnitz, Germany and [3]Fraunhofer ENAS, Chemnitz, Germany

Email: Sreetama Banerjee* - sreetama.banerjee@physik.tu-chemnitz.de;

Georgeta Salvan* - salvan@physik.tu-chemnitz.de

* Corresponding author

Additional experimental data

Fabrication and current-voltage characteristics of HED-TIE devices

The HED-TIE devices are fabricated by patterning submicrometre trenches on silicon wafers. The device has interdigitated electrodes for increasing the electrical channel volume and thus increasing the total device current. The device had channel length of ca. 100 nm and a width of 500 μm. The patterned trenches are initially etched using anisotropic etching followed by isotropic etching to form the cavity at the bottom of the trench. This allows for electrical isolation between the electrodes after the metallization step. The fabrication process in this case was similar to what is reported in [1], with some modification to control the trench geometry in a better way. The modification in the fabrication process flow will be discussed in detail in a following article. Figure S1a shows the cross-sectional scanning electron microscopic (SEM) image of a similar fabricated device, prior to the drop-coating of TIPS-pentacene solution.

Figure S1b shows the output characteristics of the fabricated device after drop-coating of TIPS-pentacene. It can be seen that the current amplitude increases when the device is exposed to ambient and measured after two days. As discussed in the main article, it can be due to the incorporation of water and oxygen molecules in the TIPS-pentacene film.

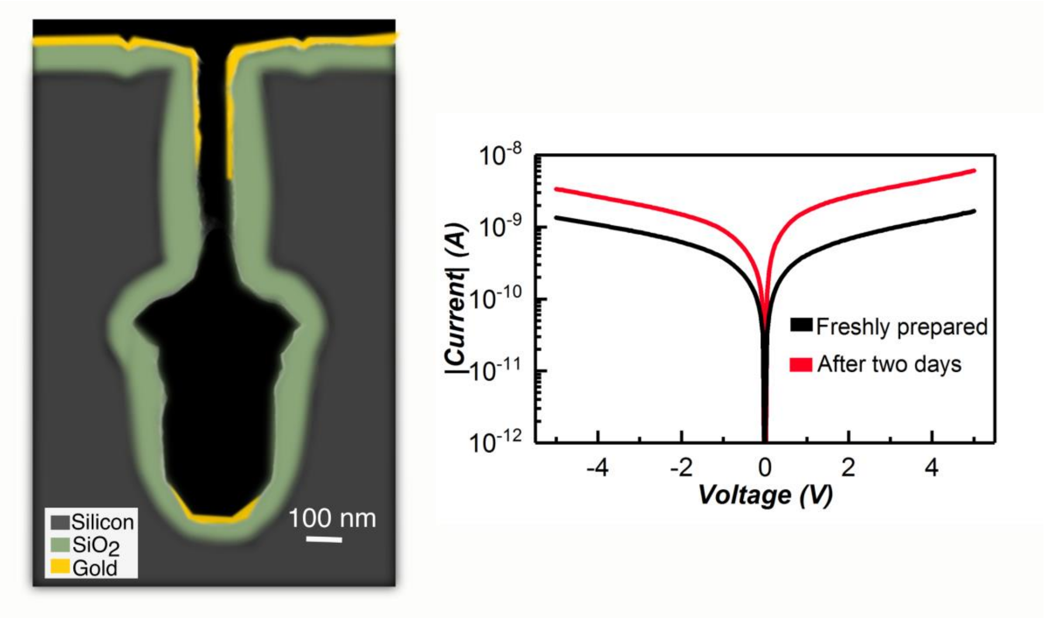

Figure S1: (a) Cross-sectional SEM image of a HED-TIE structure before TIPS-pentacene deposition. (b) Output characteristics of the TIPS-pentacene HED-TIE device, measured for freshly prepared sample and after two days of sample preparation.

Output characteristics of OFETs

Figure S2a shows the output characteristics of TIPS-pentacene OFET. p-Type conduction behaviour is observed, as expected. On the other hand, when the OFET device is prepared with TIPS-pentacene solution diluted in deionized water, the OFET characteristics (shown in Figure S2b) can be observed to degrade drastically. The current magnitude increases on a similar scale as in the HED-TIE device, when it is exposed to ambient.

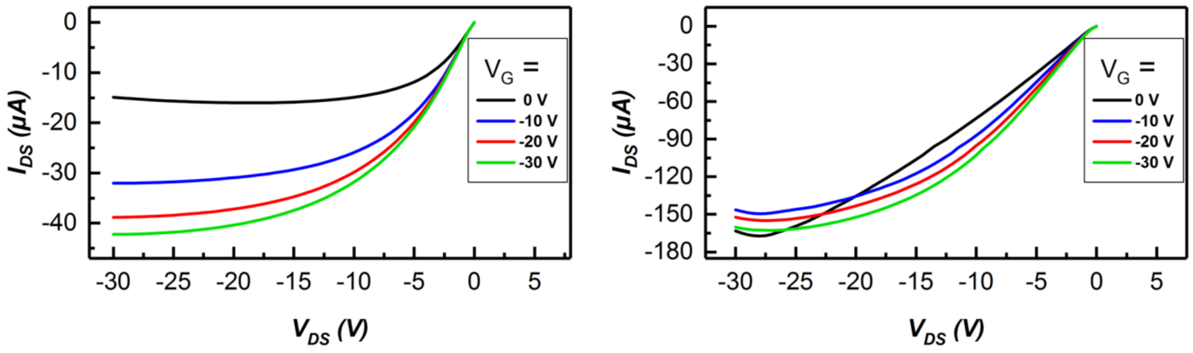

Figure S2: (a) Output characteristics of TIPS-pentacene OFET. (b) Output characteristics of an OFET prepared from the same TIPS-pentacene solution, diluted in water (1:1).

References

[1] Banerjee, S.; Bülz, D.; Solonenko, D.; Reuter, D.; Deibel, C.; Hiller, K.; Zahn, D. R. T., Salvan, G. *Nanotechnology* **2017,** *28,* 195303. doi:10.1088/1361-6528/aa6713
